# Insights from an Exploratory Retrospective Cohort Study: Are Face-to-Face Follow-Up Consultations after Colonoscopy a Thing of the Past?

**DOI:** 10.1159/000530165

**Published:** 2023-03-24

**Authors:** Jerrald Lau, Ning-Qi Pang, Chermaine Ang, Ker-Kan Tan

**Affiliations:** ^a^Yong Loo Lin School of Medicine, National University of Singapore, Singapore, Singapore; ^b^Saw Swee Hock School of Public Health, National University of Singapore, Singapore, Singapore; ^c^Department of Surgery, National University Hospital, Singapore, Singapore

**Keywords:** Colonoscopy, Healthcare sustainability, New models of care, Teleconsultation

## Abstract

**Background:**

Colonoscopy is a commonly performed procedure, but most patients will not actually be found with colorectal cancer. Subsequent face-to-face consultations to explain post-colonoscopy findings are common despite the time and cost-saving benefits of teleconsultation, especially in a post-COVID-19 era. This exploratory retrospective study examined the proportion of post-colonoscopy follow-up consultations that could have been converted to teleconsultation within a tertiary hospital in Singapore.

**Methods:**

A retrospective cohort of all patients who underwent colonoscopy in the institution from July to September 2019 was identified. All follow-up face-to-face consultations related to the index colonoscopy from the scope date to 6 months post-colonoscopy were traced. Clinical data relevant to the index colonoscopy and these consultations were extracted from electronic medical records.

**Results:**

The cohort consisted of 859 patients (68.5% male, age range: 18–96 years). Of these, 15 (1.7%) had colorectal cancer, but the majority (*n* = 643, 74.9%) were scheduled for at least one post-colonoscopy visit − a total of 884 face-to-face clinical visits. The final sample was 682 (77.1%) face-to-face post-colonoscopy visits that did not involve any procedures performed or indicated the need for any subsequent follow-up.

**Conclusion:**

If such “unnecessary” post-colonoscopy consultations exist within our institution, then similar situations possibly occur elsewhere. As COVID-19 continues to periodically tax healthcare systems worldwide, preservation of resources will remain integral alongside quality standards of routine patient care. There is a need for detailed analyses and modeling to hypothesize potential savings by also considering the start-up and maintenance costs of switching to a teleconsultation-dominated system.

## Introduction

Colonoscopy is a frequently performed lower gastrointestinal procedure in the management of colorectal cancer (CRC). While most patients undergoing colonoscopy will not actually have CRC, there will typically be a subsequent face-to-face consultation to explain post-colonoscopy findings.

Due to the COVID-19 pandemic, adoption of teleconsultation has especially been accelerated to ensure continuity of care while reducing unnecessary contact among patients [1−3]. Other benefits of teleconsultation include reduction in patient, caregiver, and institutional time and costs [4, 5]. With this in mind, it seems ironic that face-to-face consultation after a “normal” colonoscopy remains the standard of care despite the ease of evaluating symptoms and conveyance of relevant findings using alternative remote modalities. This exploratory retrospective study therefore examined the proportion of post-colonoscopy follow-up face-to-face consultations that could potentially have been converted to teleconsultation within a tertiary hospital in Singapore.

## Materials and Methods

We identified a retrospective cohort of all patients who underwent colonoscopy in our institution from July to September 2019. The timeframe was selected to predate the onset of the COVID-19 pandemic in Singapore. Ethical approval was obtained from the National Healthcare Group's Domain Specific Review Board (DSRB Ref: 2021/00936) in accordance with the Declaration of Helsinki. As the study utilized retrospective institutional data, written informed consent from participants was not required in accordance with local/national guidelines.

Using the institutional electronic medical records system, we traced all follow-up face-to-face consultations related to the index colonoscopy from the scope date to 6 months post-colonoscopy. Relevant data were extracted using a standardized data collection form, comprising the following indicators: colonoscopy diagnosis, number of clinical visits post-colonoscopy, dates of visit, purpose of visit, procedures performed, persistence of pre-colonoscopy symptoms, and the presence of new patient-reported complaints.

## Results

The cohort consisted of 859 patients (68.5% male, age range: 18–96 years). All patients were native to our institution (i.e., not referred or co-treated from/by other acute or tertiary institutions). Of these patients, 15 (1.7%) had CRC, but the majority of patients (*n* = 643, 74.9%) were scheduled for, and attended, at least one post-colonoscopy consultation − which accounted for a total of 884 face-to-face clinical visits.

Further stratification demonstrated that only 200 of the 859 patients (23.3%) in our cohort had their index colonoscopy performed for screening purposes (i.e., a positive fecal occult blood test result). Of these, 138 patients (69.0%) received a face-to-face follow-up consultation, but persistence of pre-colonoscopy symptoms and/or highlighting of new complaints was only present in 12 of these visits (8.7%). None of the 138 patients required any procedures performed in their follow-up visits.

**Fig. 1:**
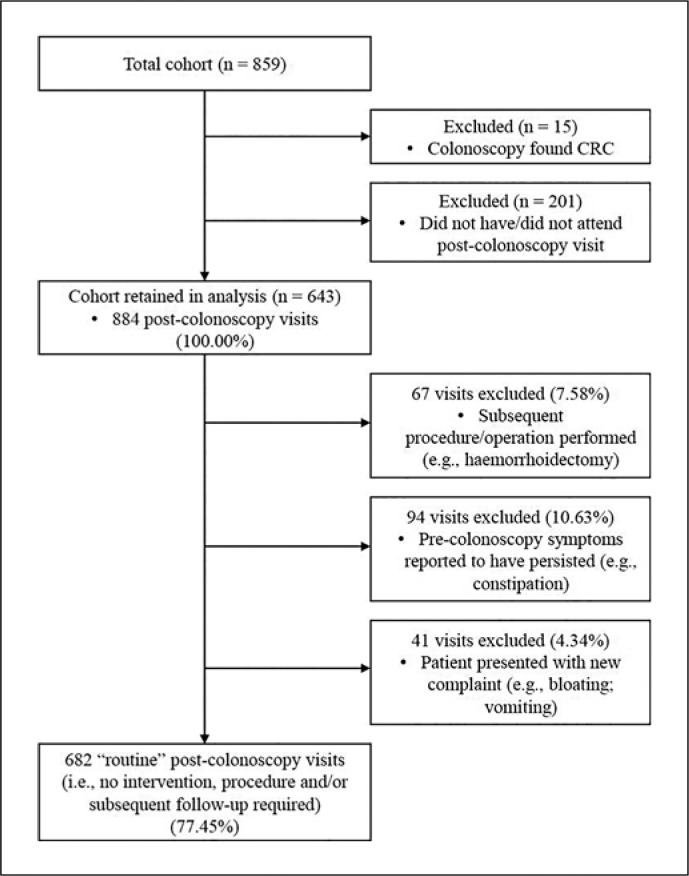
Cohort inclusion and data extraction flowchart.

To estimate the number of follow-up consultations that could have potentially been converted to teleconsultation, we applied the following eligibility criteria. First, we excluded all visits in which a subsequent procedure/operation (e.g., hemorrhoidectomy) was performed, resulting in 817 (92.4%) visits being retained in the analysis. We then excluded the 94 (10.6%) visits in which pre-colonoscopy symptoms were recorded to have persisted. Finally, we excluded the 41 (4.6%) visits in which patients highlighted a new complaint. The final sample was therefore 682 (77.1%) “routine” post-colonoscopy visits that did not involve any intervention or procedure performed nor indicated the need for any subsequent follow-up visits, in a physical face-to-face setting (Fig. 1).

## Conclusion

Our exploratory data highlight that if such “unnecessary” post-colonoscopy consultations exist within our institution, then there are possibly similar situations occurring elsewhere in the public healthcare system. These face-to-face consultations result in inconvenience and costs for patients, with the additional risks associated with travelling between home and hospital within the COVID-19 pandemic climate. Typical unsubsidized fees for an outpatient specialist repeat consultation in a public tertiary hospital in Singapore, with no procedures performed, range from US$ 67 to US$ 81 per visit [6]. While these only represent out-of-pocket costs and do not accurately capture institutional or indirect time-costing, the 682 visits could have saved our patient cohort between US$ 45,630 and US$ 55,263 had their follow-up been deemed unnecessary. This is nearly US$ 223,000 saved if extrapolated to an entire year's cohort of similar visits from just a single healthcare institution. These follow-up visits could also have been conducted using alternative means such as tele-video consultation.

Apart from patients, caregivers who take time off to accompany these patients may incur direct (e.g., transport) or indirect (e.g., loss of work productivity) costs for what is likely an inconsequential clinical visit. Healthcare institutions are also likely to incur unnecessary costs as more clinical and administrative manpower is needed to logistically facilitate a face-to-face consultation. As COVID-19 continues to periodically tax healthcare systems worldwide, the need for preservation of resources will remain integral, even as quality standards of routine patient care must be maintained.

We caveat that in contrast to scholarly discussion surrounding the recently published NordICC trial, our data should not be taken as a commentary on the benefits of colonoscopy [7, 8]. Nonetheless, we highlight that moving forward, there is a need for detailed analyses using a larger and more rigorous (potentially multicenter) cohort to confirm our findings. Modeling to evaluate the cost-effectiveness of face-to-face post-colonoscopy visits for patients without need for further procedures or follow-up complaints should also be considered, and these should account for the start-up and maintenance costs of switching to a predominantly teleconsulting system.

Patients' perceptions, barriers, and facilitators toward teleconsultation must also be known prior to widespread implementation, but healthcare costs continue to rise unsustainably in many high developmental index countries, including Singapore [9]. We urge policymakers, healthcare administrators, and physicians to overcome organizational inertia by identifying key areas in which time and costs in health services can be streamlined in an evidence-based manner.

## Acknowledgments

The authors would like to thank Si-Ying Fong and Yong Kang Chua for their support in data acquisition and extraction. They did not receive compensation for their contribution to this work.

## Statement of Ethics

Ethical approval was obtained from the National Healthcare Group's Domain Specific Review Board (DSRB Ref: 2021/00936) in accordance with the Declaration of Helsinki. As the study utilized retrospective institutional data, written informed consent from participants was not required in accordance with local/national guidelines.

## Conflict of Interest Statement

The authors have no conflicts of interest to declare.

## Funding Sources

This study was supported by the National Medical Research Council Singapore's Health Services Research Grant (HSRGMS20nov-0002) and the National University Health System's Family Medicine/Primary Care/Health Services Research Seed Grant (NUHSRO/2021/064/RO5+6/FMPCHSRG-Mar21/02). The funders had no role in the conceptualization, design, data acquisition and analysis, manuscript preparation, or decision to publish the research. The authors have no other relevant financial or nonfinancial interests to disclose.

## Author Contributions

Jerrald Lau, Ning-Qi Pang, and Ker-Kan Tan conceptualized the study. Chermaine Ang collected the data. Jerrald Lau and Chermaine Ang performed data curation. Jerrald Lau and Ning-Qi Pang performed statistical analysis of the data. Jerrald Lau drafted the initial manuscript. Ker-Kan Tan provided resources and formal supervision. All the authors critically reviewed and revised the manuscript.

## Data Availability Statement

All data generated or analyzed during this study are included in this article. Further inquiries can be directed to the corresponding author.
